# Ischemic Preconditioning Attenuates the Decline in Repeated Anaerobic Performance Under Simulated Altitude: A Randomized Crossover Study

**DOI:** 10.3390/sports13090313

**Published:** 2025-09-08

**Authors:** Miłosz Drozd, Jakub Chycki, Adam Maszczyk, Hiago L. R. Souza, Adam Zajac, Moacir Marocolo

**Affiliations:** 1 Institute of Sport Sciences, The Jerzy Kukuczka Academy of Physical Education in Katowice, 40-065 Katowice, Polanda.maszczyk@awf.katowice.pl (A.M.); 2 Department of Biophysics and Physiology, Federal University of Juiz de Fora, Juiz de Fora 36036-900, Brazil; hlrsouza@gmail.com (H.L.R.S.); moacir.marocolo@ufjf.br (M.M.)

**Keywords:** athletic performance, creatine kinase, cycling, hypoxia, oxygenation, tourniquets

## Abstract

Background: This study examined the effects of repeated ischemic preconditioning (IPC) combined with normobaric hypoxia on anaerobic performance and physiological stress markers. Methods: Fourteen physically active males (22.3 ± 3.1 years) completed three randomized, single-blind crossover sessions under the following conditions: (1) normoxia (NOR), (2) normobaric hypoxia (HYP; FiO_2_ = 14.7%), and (3) hypoxia with IPC (IPC-HYP). Each session included three 30 s cycling Wingate tests separated by four minutes of passive recovery. Blood samples were collected pre-exercise, immediately post-exercise, and 15 min post-exercise to assess lactate, pH, bicarbonate (HCO_3_^−^), and creatine kinase (CK) activity. Results: Peak power output was highest under NOR during Wingate II and III. IPC-HYP attenuated the decline in peak power compared to that under HYP (e.g., Wingate II: 15.56 vs. 12.52 W/kg). IPC-HYP induced greater lactate accumulation (peak: 15.45 mmol/L, *p* < 0.01), more pronounced acidosis (pH: 7.18 post-exercise), and lower bicarbonate (9.9 mmol/L, *p* < 0.01). CK activity, measured immediately and then 1 h and 24 h post-exercise, was highest under IPC-HYP at 24 h (568.5 U/L). Conclusions: IPC-HYP mitigates the decline in peak anaerobic power observed under hypoxia, despite eliciting greater metabolic and muscular stress. These findings suggest that IPC may enhance physiological adaptation to hypoxic training, potentially improving anaerobic performance.

## 1. Introduction

Enhancing anaerobic performance is a fundamental objective for athletes across various sport disciplines, particularly those requiring short-duration and high-intensity efforts [1,2]. Traditional training methodologies have evolved to incorporate innovative techniques such as ischemic preconditioning (IPC) and hypoxic training, both of which aim to optimize physiological adaptations beyond conventional approaches. When applied independently, these methods increase physiological stress, promoting metabolic and vascular adaptations hypothesized to enhance anaerobic performance [3,4].

IPC is a non-invasive technique involving brief cycles of ischemia and reperfusion applied to a limb before exercise; it has been shown to enhance oxygen utilization, improve blood flow dynamics, and delay fatigue onset [3,4]. Additionally, IPC has been linked to increased ATP availability, reduced oxidative stress, and improved muscle contractile efficiency [5,6], all of which contribute to greater exercise tolerance and enhanced performance in anaerobic tasks.

Hypoxic training, whether through simulated environments or natural high-altitude exposure, has been associated with improvements in both aerobic and anaerobic capacity [7,8,9]. Hypoxia induces a cascade of physiological responses, including upregulation of hypoxia-inducible factors, increased erythropoiesis, and enhanced mitochondrial efficiency [10]. While traditionally linked to endurance adaptations, emerging evidence suggests that intermittent hypoxic training may also enhance glycolytic energy pathways and buffering capacity, thereby contributing to anaerobic performance [11].

Recent studies indicate that IPC and hypoxia share overlapping physiological mechanisms, particularly in their ability to precondition the body to perform under conditions of limited oxygen availability [12,13,14], with both interventions activating protective cellular pathways such as improved oxidative metabolism, reduced lactate accumulation, and enhanced vascular reactivity [15,16]. Furthermore, both approaches may influence key biochemical and neuromuscular markers such as creatine kinase activity, acid–base balance (pH and HCO_3_^−^), and muscle fatigue dynamics, which are critical determinants of high-intensity performance capacity.

However, although both IPC and hypoxic training have been investigated in empirical studies [10,17,18], no research has specifically examined their combined effects on anaerobic performance. This is important, as their combined stressors may elicit synergistic physiological adaptations, such as heightened metabolic stress, amplified muscle fiber recruitment, and improved acid–base balance [7,19], all of which are critical determinants of anaerobic performance. Additionally, their concurrent application may affect power kinetics (e.g., time to peak power), neuromuscular fatigue (fatigue slope), and post-exercise recovery markers, including muscle damage indices.

To address this gap, we applied a repeated Wingate testing protocol under three conditions (normoxia, hypoxia, and IPC-hypoxia) to assess acute changes in performance (peak power output, time to peak power, fatigue slope, total work), physiological response (lactate, pH, HCO_3_^−^), and recovery biomarkers (creatine kinase activity). Therefore, the aim of this study was to investigate the acute effects of repeated maximal anaerobic cycling efforts under different environmental and physiological conditions on anaerobic performance parameters, biochemical markers, and muscle damage. Additionally, this study aimed to determine whether the combined application of IPC and hypoxia elicits synergistic or additive effects on performance and physiological responses compared to each intervention applied independently.

## 2. Materials and Methods

### 2.1. Participants

A priori sample estimation (G*Power 3.1.9.2, Heinrich-Heine Universität Düsseldorf, Düsseldorf, Germany; http://www.gpower.hhu.de accessed on 10 April 2024) was conducted using a three-group, repeated measures, within-subjects design with a moderate effect size (f = 0.48), α = 0.05, power (1 − β) = 0.8 and a correlation of 0.5. The analysis indicated a minimum required sample size of 12. The effect size was derived from studies included in a systematic review [18] that specifically measured power output in cycling exercise. We therefore recruited fourteen healthy physically active men (age: 22.3 ± 3.1 years; height: 180.3 ± 3.4 cm; body mass: 76.8 ± 5.2 kg; body fat: 11.8 ± 3.1%; BMI: 23.7 ± 2.56), each engaging in at least 4–6 h of structured physical activity per week (including strength or endurance training), with a minimum of three years of continuous training experience. The eligibility criteria included being male, being a non-smoker, disclosing no cardiovascular or metabolic disease, being normotensive (<140/90 mmHg and not using antihypertensive medication), having no history of performance-enhancing drug use (androgenic anabolic steroids), being physically healthy (no recent musculoskeletal injury) and having no experience of using a pneumatic occlusion maneuver. We exclusively recruited male participants due to performance variations observed during different phases of the menstrual cycle [20]. Participants were instructed to maintain their regular sleep patterns and dietary habits throughout the study while refraining from the use of stimulants, supplements, and other ergogenic aids. To control for circadian rhythm variations, all testing sessions were conducted at the same time of day (9:00–11:00). Additionally, participants were required to abstain from high-intensity exercise for at least 72 h before testing to minimize any residual fatigue effects.

Before enrollment, all participants were informed about the study’s objectives, potential risks, and experimental procedures. Written informed consent was obtained from all subjects prior to participation. The study protocol was approved by the Local Bioethics Committee for Scientific Research (approval number 02/2019) and conducted in accordance with the ethical standards outlined in the Declaration of Helsinki.

### 2.2. Experimental Design

**Figure 1:**
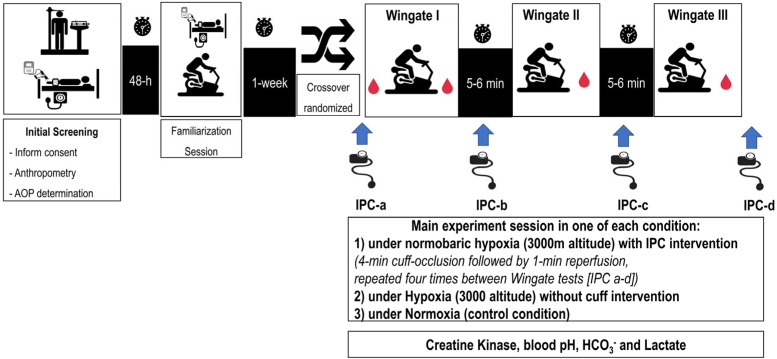
Schematic representation of the study design. AOP: arterial occlusion pressure; IPC: ischemic preconditioning.

We used a single-blind crossover design (i.e., the researchers were blinded to the protocol intervention received by each participant), in which participants served as their own controls and completed three experimental sessions in a randomized order, each consisting of three 30 s cycling Wingate tests under the following conditions: (1) a control session under normoxia (NOR), (2) an experimental session under normobaric hypoxia at a simulated altitude of 3000 m above sea level (HYP), and (3) an experimental session under hypoxia after IPC cuff protocol (IPC-HYP). To further investigate performance dynamics, the time to peak power (TPP), relative peak power output (PPO), fatigue slope, and total work were extracted from each Wingate test using Lode Ergometry Manager software (LEM 10, Groningen, The Netherlands), allowing for detailed kinetic analysis across trials (Figure 1).

The exercise protocol was conducted under normobaric hypoxia using the LOW OXYGEN^®^ climate system (LOW OXYGEN^®^ SYSTEMS, Berlin, Germany), which continuously monitored and maintained conditions corresponding to an altitude of 3000 m above sea level (fraction of inspired oxygen [FiO_2_] of 14.7%). FiO_2_ was controlled regularly with an electronic oximeter (PC-900, MDI Consultants, Toronto, ON, Canada), with a variation within ± 2%. Participants were not provided with any information regarding the FiO_2_ level for any trial. However, they were informed that the level of hypoxia would remain consistent across all sessions.

To assess the acute effects of repeated Wingate tests under the three different conditions, the following physiological markers were evaluated: capillary blood lactate concentration, blood pH, bicarbonate (HCO_3_^−^), and serum creatine kinase (CK) activity. CK activity, as an indicator of muscle damage, was assessed at baseline and at three time points post-exercise: immediately after, 1 h post-exercise, and 24 h into recovery. Blood lactate concentration and acid–base balance were measured at rest, at three minutes after each Wingate test, and at the tenth minute of recovery following the final Wingate test.

### 2.3. Thirty-Second Wingate Test

Three standardized 30 s lower-limb Wingate tests were performed using a Lode Excalibur Sport cycle ergometer (Lode Medical Technology, Groningen, The Netherlands). Each test consisted of maximal pedaling exercise with a load equivalent to 7.5% of body mass, which is the standardized protocol for Wingate anaerobic tests and has been validated for untrained to moderately trained male populations. This choice ensures comparability with previous literature and provides optimal resistance to elicit peak anaerobic power. Before each test, participants completed a warm-up consisting of 5 min of pedaling at 60 rpm with a load of 1.5 kg, followed by 2 × 3–4 s bouts of maximal pedaling with loads equivalent to 5% and 7% of their body mass. This was followed by 5–6 min of dynamic stretching and 5 min of passive rest. The ergometer’s torque factor and data sampling rate were configured to enable second-by-second power output recording, which was essential for calculating TPP and fatigue slope.

TPP was defined as the time elapsed from the start of the sprint to the point of maximum power output.

Fatigue slope was calculated as the average rate of decline in power output from the point of peak power to the end of the 30 s effort, according to the standard Wingate methodology. Thus, the formula:

Fatigue slope = (Peak Power − Final Power)/(Time from Peak Power to 30 s)

expresses the decrement occurring specifically after the attainment of PPO until the 30 s mark, rather than over the entire test duration.

This approach quantifies the magnitude of fatigue-induced power loss per second over the latter portion of the test, which is a standard method in Wingate analysis and is consistent with previous literature [2]. If your intention was to express the fatigue slope over the entire 30 s test, please clarify, but the above method, using the interval from peak power to the end of the test, is correct and widely accepted.

### 2.4. Ischemic Preconditioning

Each IPC application was performed bilaterally in a supine rest position and consisted of one cycle of 4 min occlusion followed by 1 min reperfusion. The IPC protocol was applied immediately before the first Wingate test and immediately after each subsequent test, with reperfusion occurring 60 s before the start of each Wingate test. IPC was also performed after the last Wingate test, totaling four IPC applications. Manual pneumatic cuffs (Leg Cuffs V3.1, FitCuffs^®^ device; Odder, Denmark, 11 cm width, 80 cm length) were placed on the proximal thigh and inflated to each participant’s individual arterial occlusion pressure (AOP).

To determine each participant’s AOP, the cuff device was applied to the most proximal portion of the dominant thigh. Following a general warm-up and a 5 min rest period, the full arterial occlusion pressure (100% AOP) was measured in a supine position using an Edan SD3 Doppler device equipped with a 2 MHz probe and OLED screen (Edan Instruments, Shenzhen, China). AOP was assessed twice, with a 5 min interval between measurements, following established protocols [4,5]. The average of the two values used for analysis. The calculated average 100% AOP used for the intervention was 188 ± 19 mmHg.

### 2.5. Body Mass and Body Composition Evaluation

Anthropometric assessments were conducted between 08:00 and 09:00 under standardized conditions. Participants consumed their last meal at 20:00 the previous day and hydrated with 1.5 L of water between 20:00 and 22:00. They arrived at the laboratory after an overnight fast, having abstained from exercise for 24 h and avoided alcohol, caffeine-containing beverages, and carbohydrate-rich liquids before testing.

Body mass (BM) and composition were measured using bioelectrical impedance analysis (InBody 370, InBody USA, Cerritos, CA, USA). The assessed parameters included body fat percentage (FAT), fat-free mass (FFM), and total body water (TBW), further divided into intracellular (ICW) and extracellular (ECW) compartments. The body mass index (BMI) was calculated from body mass and height measurements.

### 2.6. Biochemical Analysis

Capillary blood samples were collected from the fingertip using a disposable Medlance^®^ Special lancet (0.8 mm blade, 2.0 mm penetration depth). Approximately 55 μL of blood was collected into a heparinized capillary tube for the determination of blood lactate (La) concentrations (mml/L), bicarbonate (HCO_3_^−^) levels (mEq/L), and pH values using a blood gas analyzer (Biosen C-line Clinic, EKF-diagnostic GmbH, Barleben, Germany).

Venous blood samples for CK activity analysis (U/L) were collected from the antecubital vein in a seated position at rest (15 min before the first Wingate test) and at the 1 h and 24 h recovery time points. Each sample consisted of 10 mL of blood, 8 mL collected into a tube without anticoagulant and 2 mL collected into a tube containing EDTA, using the Vacutainer technique. Serum was obtained via centrifugation (2000× *g*, 4 °C, 15 min) and stored at −20 °C for subsequent biochemical analysis. CK activity was determined using a Randox Laboratories diagnostic kit (Randox Laboratories, Crumlin, UK). The intra- and inter-assay coefficients of variation (CV) were 1.93% and 3.63%, respectively.

In addition to standard acid–base markers, CK was selected as a marker of exercise-induced muscle damage due to its delayed post-exercise elevation, enabling the evaluation of tissue stress and recovery across a 24 h window. All blood gas and lactate samples were collected within a consistent 30–45 s window following each Wingate test to minimize timing bias.

### 2.7. Statistical Analysis

The normality of the data distribution was assessed using the Shapiro–Wilk test for each condition (NOR, HYP, IPC-HYP) at each time point. Data were assumed to follow a normal distribution if *p* > 0.05. Sphericity was evaluated using Mauchly’s test for repeated-measures ANOVA. In cases where sphericity was violated (common when more than two factor levels are involved), Huynh–Feldt or Greenhouse–Geisser corrections were applied. A two-way repeated-measures ANOVA was performed to assess the effects of condition (NOR, HYP, IPC-HYP) and time/trial (Wingate I–III) on the measured variables. This approach allowed us to evaluate the following: (i) the main effect of condition (whether differences exist among NOR, HYP, and IPC-HYP), (ii) the main effect of time/trial (whether performance or biochemical markers changed over successive Wingate tests), and (iii) the interaction effect between condition and time/trial (whether changes over time differed across conditions).

If significant main effects or interactions were detected, Bonferroni post hoc tests were conducted to identify specific pairwise differences among conditions (NOR vs. HYP, NOR vs. IPC-HYP, HYP vs. IPC-HYP) and across time points (Wingate I vs. II vs. III), controlling for the increased risk of Type I error. All statistical analyses were conducted using Statistica software (version 13, StatSoft, Tulsa, OK, USA), with a significance level set at α = 0.05. Data are presented as means ± SD.

## 3. Results

### 3.1. Time to Peak Power (TPP)

Mean TPP values across Wingate tests I–III for each experimental condition are presented in Table 1. A two-way repeated-measures ANOVA revealed a significant main effect of condition [F(2,18) = 8.22, *p* = 0.003, η^2^ = 0.48], repetition [F(2,18) = 4.53, *p* = 0.025, η^2^ = 0.33], and a significant condition × repetition interaction [F(4,36) = 5.01, *p* = 0.003, η^2^ = 0.36].

Post hoc analysis indicated that in Wingate I, TPP was significantly longer in the IPC-HYP condition (2.71 ± 0.14 s) compared to under NOR (2.27 ± 0.09 s; *p* < 0.01). This effect was magnified in Wingate II and III, where TPP values in IPC-HYP (4.37 ± 1.15 s and 3.84 ± 0.34 s, respectively) were significantly greater than under both HYP and NOR (*p* < 0.001).

### 3.2. Relative Peak Power Output (PPO)

Mean relative PPO values (W/kg) for Wingate I–III are summarized in Table 1. The ANOVA revealed significant main effects of condition [F(2,18) = 15.10, *p* < 0.001, η^2^ = 0.63] and repetition [F(2,18) = 9.54, *p* = 0.002, η^2^ = 0.51], as well as a significant interaction [F(4,36) = 4.80, *p* = 0.004, η^2^ = 0.35].

During Wingate II, PPO was significantly higher under NOR (17.96 ± 1.26 W/kg) compared to under both HYP (12.52 ± 2.13 W/kg; *p* < 0.001) and IPC-HYP (15.56 ± 1.68 W/kg; *p* = 0.02), with that under IPC-HYP also exceeding that under HYP (*p* = 0.03). In Wingate III, PPO was highest under NOR (16.62 ± 1.13 W/kg), while it was significant greater under IPC-HYP (14.58 ± 1.22 W/kg) than under HYP (12.65 ± 1.08 W/kg; *p* = 0.03). However, the value for NOR was not significantly different from that for HYP in Wingate III, as indicated by the table notations. No significant differences were observed in Wingate I.

### 3.3. Fatigue Slope

Fatigue slope values (W/s) across all Wingate efforts are detailed in Table 1. Repeated-measures ANOVA indicated main effects of condition [F(2,18) = 6.80, *p* = 0.006, η^2^ = 0.43] and repetition [F(2,18) = 5.10, *p* = 0.017, η^2^ = 0.36], and a significant condition × repetition interaction [F(4,36) = 3.02, *p* = 0.03, η^2^ = 0.25].

NOR consistently demonstrated the highest fatigue slope across all three Wingate tests. In Wingate II, the fatigue slope under NOR (38.38 ± 2.31 W/s) was significantly higher than that under HYP (25.44 ± 1.12 W/s; *p* < 0.001) and IPC-HYP (35.41 ± 1.86 W/s; *p* = 0.04), with that under IPC-HYP also being higher than that under HYP (*p* < 0.01). In Wingate I and III, similar patterns were observed, though differences did not reach statistical significance.

### 3.4. Total Work (TW)

As shown in Table 1, total work (J/kg) differed significantly by condition [F(2,18) = 7.90, *p* = 0.003, η^2^ = 0.47] and repetition [F(2,18) = 14.22, *p* < 0.001, η^2^ = 0.61], but not for the interaction [F(4,36) = 2.10, *p* = 0.10].

In Wingate II and III, TW was significantly higher under NOR compared to both HYP and IPC-HYP (*p* < 0.01). IPC-HYP also yielded significantly greater TW than HYP (*p* < 0.05). No significant differences were observed in Wingate I.

### 3.5. Blood Lactate, pH and Bicarbonate Concentration

Descriptive statistics for lactate, pH, and HCO_3_^−^ at rest, during exercise (Wingate I–III), and 10 min post-exercise are shown in Table 2.

A two-way repeated-measures ANOVA for lactate concentration indicated significant effects of condition [F(2,18) = 16.28, *p* < 0.001, η^2^ = 0.64], time [F(4,36) = 96.44, *p* < 0.001, η^2^ = 0.91], and the condition × time interaction [F(8,72) = 5.98, *p* < 0.01, η^2^ = 0.40].

By Wingate II, both HYP and IPC-HYP showed higher lactate concentrations than NOR (*p* < 0.05). In Wingate III, the lactate concentration of IPC-HYP (14.67 ± 1.02 mmol/L) exceeded that of NOR (*p* < 0.01), but the difference between IPC-HYP and HYP did not reach statistical significance (*p* = 0.09), as indicated in Table 2. At 10 min post-exercise, IPC-HYP showed the highest lactate concentration (15.45 ± 2.20 mmol/L), significantly greater than that of NOR (*p* < 0.001) and HYP (*p* < 0.01). Resting values did not differ between conditions.

Resting pH levels were similar across groups. A progressive decline in pH was observed during Wingate I–III, with IPC-HYP showing the most pronounced acidosis, particularly post-Wingate II (7.23 ± 0.03) and III (7.18 ± 0.03). Post hoc analysis confirmed that the pH in IPC-HYP was significantly lower than that in both NOR and HYP after Wingate II and III (*p* < 0.01), as denoted by the table notations. At 10 min post-exercise, the pH in IPC-HYP remained significantly lower than that in NOR and HYP (*p* < 0.01).

Bicarbonate levels also declined across Wingate bouts. IPC-HYP demonstrated the lowest HCO_3_^−^ levels, particularly after Wingate III (11.3 ± 0.56 mmol/L) and 10 min post-exercise (9.9 ± 0.45 mmol/L), with both values significantly lower than those in HYP and NOR (*p* < 0.01), as indicated in Table 2.

### 3.6. Creatine Kinase Activity (CK)

CK values at rest and at 1 h and 24 h post-exercise are presented in Table 3. Repeated-measures ANOVA showed main effects of condition [F(2,18) = 8.60, *p* = 0.002, η^2^ = 0.49], time [F(2,18) = 25.40, *p* < 0.001, η^2^ = 0.74], and a condition × time interaction [F(4,36) = 6.14, *p* = 0.001, η^2^ = 0.41].

While no significant differences were detected at rest, 1 h post-exercise CK activity was significantly elevated under HYP (309.6 ± 55.4 U/L) compared to NOR (258.4 ± 36.2 U/L; *p* = 0.02), with IPC-HYP trending similarly (*p* = 0.05 vs. NOR). At 24 h, IPC-HYP demonstrated the highest CK activity (568.5 ± 187.4 U/L), significantly higher than both HYP (*p* = 0.01) and NOR (*p* < 0.001), indicating greater muscle membrane disruption.

## 4. Discussion

The present study investigated the effects of IPC and hypoxia on anaerobic performance, metabolic stress, and muscle damage markers. We initially hypothesized that hypoxia would impair anaerobic performance, reflected by a reduced peak power and total work, compared to normoxia, and that IPC applied prior to hypoxia could attenuate these performance decrements. Additionally, based on the previous literature suggesting a protective role of IPC, we hypothesized that IPC-HYP might reduce metabolic stress and muscle damage typically associated with hypoxic exercise. To further elucidate neuromuscular responses, we also examined power output kinetics, including the time to peak power and fatigue slope. Contrary to our initial expectations, IPC-HYP was associated with greater metabolic stress (elevated blood lactate concentrations) and increased muscle damage (higher CK activity) compared to both NOR and HYP. Nevertheless, IPC-HYP effectively mitigated the decline in anaerobic performance observed under hypoxia alone, suggesting that IPC may enhance performance tolerance despite heightened physiological strain.

Our results demonstrated that NOR consistently led to the highest PPO and total work across all Wingate trials. These findings align with the previous literature indicating that acute hypoxia impairs anaerobic performance due to reduced oxygen availability and limitations in phosphocreatine resynthesis [11]. The decrease in PPO and total work observed in the HYP condition supports this, suggesting a diminished capacity for ATP production via anaerobic glycolysis. Similarly, Bowtell et al. [21] reported marked impairments in sprint performance under acute hypoxia, which is consistent with our observation of reduced PPO and total work in the HYP condition. The partial preservation of peak power under IPC-HYP, however, indicates a modulating effect not observed under hypoxia alone. This is also in line with previous reports demonstrating improved time–trial performance at moderate altitudes following IPC [22,23,24]. Nevertheless, while those studies described reduced metabolic strain, our results revealed greater lactate accumulation, suggesting that the ergogenic effects of IPC may be strongly context-dependent. Mechanistically, such improvements have been attributed to enhanced muscle oxygen utilization and increased blood perfusion upon reperfusion. Importantly, IPC did not fully restore performance to NOR levels, indicating that its ergogenic impact may be insufficient to overcome the physiological limitations imposed by hypoxia. Moreover, TPP was significantly prolonged under IPC-HYP, particularly in later sprints, suggesting delayed force generation under combined physiological stress.

Post-exercise blood lactate levels were significantly higher under IPC-HYP than under both NOR and HYP, particularly at the 10 min post-exercise mark. This suggests an increased reliance on anaerobic glycolysis during exercise, which may result from enhanced muscle perfusion following IPC-induced vasodilation [22]. While previous studies have shown IPC to improve repeated sprint ability and power output [23,24], our results indicate that this performance enhancement may come at the cost of greater metabolic stress. Elevated lactate levels under IPC-HYP could also be attributed to a potential mismatch between oxygen delivery and utilization, leading to increased glycolytic activity despite improved muscle perfusion [25].

IPC-HYP caused the most disruptions in pH and bicarbonate levels, showing significant acid–base imbalance. HYP also reduced pH compared to NOR, but less so than IPC-HYP. This indicates that IPC-HYP increases hydrogen ions, worsening metabolic acidosis, which affects exercise performance and recovery. Fatigue slope analysis showed lower fatigue under HYP compared to NOR and IPC-HYP, indicating that participants experienced less fatigue under the HYP condition. IPC-HYP showed intermediate results, balancing peak power and metabolic fatigue.

The CK response further highlights the physiological cost associated with IPC-HYP. While NOR exhibited the lowest CK levels at all time points, IPC-HYP resulted in significantly greater CK elevations at 24 h post-exercise, surpassing those in both the NOR and HYP conditions. This suggests that IPC may exacerbate muscle fiber disruption due to enhanced mechanical stress during exercise. Previous studies have reported conflicting results regarding IPC’s influence on muscle damage, with some indicating a protective effect via ischemic tolerance [26], while others suggest increased susceptibility to damage due to augmented metabolic demand [27]. Our findings support the latter, implying that the enhanced anaerobic contribution observed in IPC-HYP may have led to greater mechanical strain on the muscle fibers, resulting in heightened CK release.

The potential synergy between IPC and hypoxia may stem from their mutual influence on mitochondrial efficiency and tissue oxygenation, as both strategies enhance the body’s ability to function with limited oxygen by improving how cells produce energy and utilize oxygen. This suggests that combining these strategies could improve their respective benefits, leading to greater improvements in anaerobic performance. However, the associated increase in muscle damage and metabolic acidosis suggests a need for caution in applying this combined strategy in repeated or chronic protocols without sufficient recovery monitoring.

From a practical standpoint, these findings suggest that IPC may serve as a useful preconditioning strategy for athletes competing in hypoxic or high-altitude environments to mitigate performance decrements. However, the concurrent rise in metabolic stress and muscle damage underscores the need for careful application, adequate recovery, and individualized monitoring.

While our research has important implications for research, they must be interpreted in light of several limitations. First, this study did not include direct muscle oxygenation measurements, which would have provided deeper insights into the physiological mechanisms underlying the observed performance and metabolic responses. Second, our sample consisted solely of untrained individuals, limiting generalizability of the findings to trained athletes. Third, as women were not included in the sample, the applicability of the results to female populations remains uncertain. Additionally, future studies should assess whether chronic IPC exposure induces adaptations that mitigate its acute metabolic stress and muscle damage effects. The inclusion of measures such as near-infrared spectroscopy (NIRS) or muscle biopsies would help clarify the mechanistic basis of the observed changes in power output, fatigue, and biochemical markers. In addition, although our study design did not allow us to directly investigate mechanistic pathways, previous work suggests several plausible explanations for the modulating effect of IPC in hypoxia. IPC may enhance muscle perfusion through improved endothelial function and vasodilation, may augment oxygen utilization through enhanced mitochondrial efficiency, and may increase glycolytic enzyme activity, thereby supporting anaerobic ATP resynthesis when oxygen availability is limited. These pathways could account for the partial performance preservation observed in our IPC-HYP condition, albeit with an associated increase in lactate accumulation and CK release. Future mechanistic studies should therefore combine IPC with muscle oxygenation measurements (e.g., NIRS) to clarify how such processes contribute to modifications in performance under hypoxic stress.

Future studies should investigate chronic training interventions combining IPC and hypoxia, explore sex-based differences, and include direct measures of muscle oxygenation (e.g., NIRS) to elucidate underlying mechanisms of adaptation. Longitudinal designs with elite athletes may further clarify whether repeated IPC exposure enhances adaptive responses without exacerbating muscle damage.

## 5. Conclusions

In summary, IPC partially mitigated the decline in anaerobic performance typically observed under hypoxic conditions, as evidenced by the preserved peak power output, reduced fatigue slope, and improved total work across repeated Wingate tests. These findings support IPC as a potentially effective strategy for maintaining high-intensity exercise capacity in oxygen-restricted environments. However, the concurrent increase in metabolic stress (elevated blood lactate, reduced pH and bicarbonate) and the greater muscle damage (elevated CK levels) observed under IPC-HYP highlight the substantial physiological costs of this intervention. Therefore, while IPC may enhance acute performance under hypoxia, its application should be carefully monitored, particularly in repeated or prolonged protocols where recovery and adaptation dynamics play a critical role.

## Figures and Tables

**Table 1:** Anaerobic performance outcomes under normoxia and hypoxic conditions (values are presented as mean ± SD).

Variable	Condition	Wingate I	Wingate II	Wingate III
Time to peak power (s)	NOR	2.27 ± 0.09	2.76 ± 0.08	2.25 ± 0.11
	HYP	2.55 ± 0.09	2.45 ± 0.09	1.82 ± 0.08
	IPC-HYP	2.71 ± 0.14 ^a^	4.37 ± 1.15 ^b^	3.84 ± 0.34 ^c^
Relative peak power output (W/kg)	NOR	18.37 ± 2.05	17.96 ± 1.26 ^d^	16.62 ± 1.13
	HYP	16.76 ± 1.32	12.52 ± 2.13	12.65 ± 1.08
	IPC-HYP	17.92 ± 1.83	15.56 ± 1.68 ^e^	14.58 ± 1.22 ^f^
Fatigue slope (W/s)	NOR	39.76 ± 1.05	38.38 ± 2.31 ^g^	37.65 ± 2.03
	HYP	34.58 ± 1.63	25.44 ± 1.12	26.45 ± 1.58
	IPC-HYP	38.24 ± 2.06	35.41 ± 1.86 ^h^	34.80 ± 2.36
Total work (J/kg)	NOR	263.19 ± 12.63	257.46 ± 11.32 ^i^	213.99 ± 8.52 ^j^
	HYP	262.82 ± 10.86	214.53 ± 9.32	205.13 ± 8.03
	IPC-HYP	259.50 ± 16.36	225.52 ± 10.46 ^k^	210.04 ± 9.16 ^l^

NOR: control session under normoxia; HYP: experimental session under normobaric hypoxia at a simulated altitude of 3000 m above sea level; IPC-HYP experimental session under hypoxia after ischemic preconditioning cuff protocol. ^a^ denotes a significant difference between IPC-HYP and NOR; ^b^ denotes a significant difference between IPC-HYP and both HYP and NOR; ^c^ denotes a significant difference between IPC-HYP and both HYP and NOR; ^d^ denotes a significant difference between NOR and both HYP and IPC-HYP; ^e^ denotes a significant difference between IPC-HYP and HYP (the study did not include direct muscle oxygenation). ^f^ denotes a significant difference between IPC-HYP and HYP; ^g^ denotes a significant difference between NOR and both HYP and IPC-HYP; ^h^ denotes a significant difference between IPC-HYP and HYP; ^i^ denotes a significant difference between NOR and both IPC-HYP and HYP; ^j^ denotes a significant difference between NOR and both IPC-HYP and HYP; ^k^ denotes a significant difference between IPC-HYP and HYP; ^l^ denotes a significant difference between IPC-HYP and HYP.

**Table 2:** Lactate concentration and acid–base equilibrium variables during different exercise conditions (values are presented as mean ± SD).

Variable		NOR	HYP	IPC-HYP
Lactate	Rest	1.09 ± 0.20	1.89 ± 0.19	1.06 ± 0.29
	Wingate I	9.12 ± 0.57	8.95 ± 1.70	9.66 ± 0.34
	Wingate II	10.85 ± 1.46 ^a^	12.12 ± 2.00	10.98 ± 1.03
	Wingate III	12.36 ± 2.03	13.81 ± 1.45	14.67 ± 1.02 ^b^
	10’ Post Wingate	10.23 ± 1.61	11.08 ± 1.58	15.45 ± 2.20 ^c^
pH	Rest	7.42 ± 0.06	7.39 ± 0.05	7.41 ± 0.04
	Wingate I	7.33 ± 0.05	7.30 ± 0.06	7.28 ± 0.04
	Wingate II	7.25 ± 0.04	7.25 ± 0.04	7.23 ± 0.03
	Wingate III	7.22 ± 0.02	7.19 ± 0.02	7.18 ± 0.03
	10’ Post Wingate	7.19 ± 0.03	7.14 ± 0.01	7.12 ± 0.02 ^d^
HCO_3_^−^	Rest	24.3 ± 1.10	25.4 ± 1.30	25.2 ± 0.91
	Wingate I	18.5 ± 0.77	17.3 ± 0.81	16.3 ± 072
	Wingate II	16.2 ± 0.80	14.4 ± 0.61	13.2 ± 0.62
	Wingate III	14.1 ± 0.70	12.5 ± 0.56	11.3 ± 0.56 ^e^
	10’ Post Wingate	12.3 ± 0.61	10.3 ± 1.01	9.9 ± 0.45 ^f^

NOR: control session under normoxia; HYP: experimental session under normobaric hypoxia at a simulated altitude of 3000 m above sea level; IPC-HYP experimental session under hypoxia after ischemic preconditioning cuff protocol. ^a^ denotes a significant difference between NOR and both IPC-HYP and HYP; ^b^ denotes a significant difference between IPC-HYP and NOR; ^c^ denotes a significant difference between IPC-HYP and both NOR and HYP; ^d^ denotes a significant difference between IPC-HYP and both NOR and HYP; ^e^ denotes a significant difference between IPC-HYP and both NOR and HYP; ^f^ denotes a significant difference between IPC-HYP and both NOR and HYP.

**Table 3:** Creatine kinase activity during different exercise conditions (values are presented as mean ± SD).

Variable		NOR	HYP	IPC-HYP
Creatine Kinase	Rest	128.3 ± 40.2	149.4 ± 63.8	143.4 ± 51.3
	1 h	258.4 ± 36.2	309.6 ± 55.4 ^a^	305.2 ± 89.6
	24 h	338.8 ± 55.2	421.3 ± 87.5	568.5 ± 187.4 ^b^

NOR: control session under normoxia; HYP: experimental session under normobaric hypoxia at a simulated altitude of 3000 m above sea level; IPC-HYP experimental session under hypoxia after ischemic preconditioning cuff protocol. ^a^ denotes a significant difference between HYP and NOR; ^b^ denotes a significant difference between IPC-HYP and both NOR and HYP.

## Data Availability

The original contributions presented in the study are included in the article, further inquiries can be directed to the corresponding author/s.

## Abbreviations

The following abbreviations are used in this manuscript:

AOP	Arterial Occlusion Pressure
ANOVA	Analysis of Variance
ATP	Adenosine Triphosphate
BM	Body Mass
BMI	Body Mass Index
BFR	Blood Flow Restriction
CK	Creatine Kinase
CON	Control Condition
CV	Coefficient of Variation
ECW	Extracellular Water
FFM	Fat-Free Mass
FiO_2_	Fraction of Inspired Oxygen
HCO_3_^−^	Bicarbonate
HIPC	Hypoxic–Ischemic Preconditioning
HPC	Hypoxic Preconditioning
HYP	Normobaric Hypoxia
ICW	Intracellular Water
IPC	Ischemic Preconditioning
IPC-HYP	Ischemic Preconditioning with Hypoxia
La	Lactate
NOR	Normoxia
pH	Hydrogen Ion Concentration
PPO	Peak Power Output
RPE	Rating of Perceived Exertion
SD	Standard Deviation
SpO_2_	Peripheral Oxygen Saturation
TBW	Total Body Water
TPP	Time to Peak Power
TW	Total Work
TSI	Tissue Saturation Index
UT	Untrained Participants
Wingate	Wingate Anaerobic Test
LEM	Lode Ergometry Manager
NIRS	Near-Infrared Spectroscopy
SD3	Edan SD3 Doppler Device
G*Power	G*Power Statistical Software
